# Combined *Sabal* and *Urtica* Extracts (WS^®^ 1541) Exert Anti-proliferative and Anti-inflammatory Effects in a Mouse Model of Benign Prostate Hyperplasia

**DOI:** 10.3389/fphar.2019.00311

**Published:** 2019-03-29

**Authors:** Natascha Pigat, Edouard Reyes-Gomez, Florence Boutillon, Stefano Palea, Nicolas Barry Delongchamps, Egon Koch, Vincent Goffin

**Affiliations:** ^1^PRL/GH Pathophysiology Laboratory, Institut Necker Enfants Malades, Unit 1151, Inserm, Paris, France; ^2^Faculté de Médecine, Université Paris Descartes, Paris, France; ^3^Unité d’Histologie et d’Anatomie Pathologique, Laboratoire d’Anatomo-Cytopathologie, Biopôle Alfort, Ecole Nationale Vétérinaire d’Alfort, Maisons-Alfort, France; ^4^Inserm, U955 – IMRB, Ecole Nationale Vétérinaire d’Alfort, UPEC, Maisons-Alfort, France; ^5^Humana Biosciences SAS, Labège, France; ^6^Urology Department, Hôpital Cochin, Assistance Publique Hôpitaux de Paris, Paris, France; ^7^Dr. Willmar Schwabe GmbH & Co. KG, Karlsruhe, Germany

**Keywords:** WS^®^ 1541, BPH, inflammation, cytokines, chemokines, COX-2, iNOS, lower urinary tract syndrome (LUTS)

## Abstract

WS^®^ 1541 is a phytopharmaceutical drug combination containing a lipophilic extract from fruits of *Sabal serrulata* (WS^®^ 1473) and an aqueous ethanolic extract from roots of *Urtica dioica* (WS^®^ 1031). It is approved in several countries worldwide for the treatment of lower urinary tract syndrome (LUTS) linked to benign prostate hyperplasia (BPH). Clinical studies have demonstrated the efficacy of this unique combination in the treatment of BPH-related LUTS. However, its mechanisms of action *in vivo* remain partly uncharacterized. The aim of this study was to take advantage of a validated mouse model of BPH to better characterize its growth-inhibitory and anti-inflammatory properties. We used the probasin–prolactin (Pb-PRL) transgenic mouse model in which prostate-specific overexpression of PRL results in several features of the human disease including tissue hypertrophy, epithelial hyperplasia, increased stromal cellularity, inflammation, and LUTS. Six-month-old heterozygous Pb-PRL male mice were randomly distributed to five groups (11–12 animals/group) orally treated for 28 consecutive days with WS^®^ 1541 (300, 600, or 900 mg/kg/day), the 5α-reductase inhibitor finasteride used as reference (5 mg/kg/day) or vehicle (olive oil 5 ml/kg/day). Administration of WS^®^ 1541 was well tolerated and caused a dose-dependent reduction of prostate weight (vs. vehicle) that was statistically significant at the two highest doses. This effect was accompanied by a reduction in prostate cell proliferation as assessed by lower Ki-67 expression (qPCR and immunohistochemistry). In contrast, finasteride had no or only a mild effect on these parameters. The growth-inhibitory activity of WS^®^ 1541 was accompanied by a strong anti-inflammatory effect as evidenced by the reduced infiltration of cells expressing the leukocyte common antigen CD45. In sharp contrast, finasteride significantly increased the prostate inflammatory status according to this readout. Molecular profiling (qPCR) of 23 selected pro-inflammatory genes confirmed the strong anti-inflammatory potency of WS^®^ 1541 compared to finasteride. Since treatment of WS^®^ 1541 did not interfere with transgene expression and activity in the prostate of Pb-PRL mice, the effects observed in this study are entirely attributable to the intrinsic pharmacological action of the drug combination.

## Introduction

Benign prostate hyperplasia (BPH) is a common urological condition caused by the non-malignant enlargement of the prostate gland as men get older, and potentially leading to lower urinary tract symptoms (LUTS) and complications. Approximately one-third of men older than 40 years will report bother related to LUTS (Speakman et al., 2015) and be proposed for a treatment. At the histological level, BPH is characterized by hyperproliferation of both stromal and glandular cell compartments in the transition zone and periurethral areas of the prostate gland (Lee et al., 2011). Hence, medical treatment of LUTS intend to reduce cellular proliferation by inhibiting 5α-reductase enzymes involved in the conversion of testosterone into its active metabolite dihydrotestosterone (DHT), to decrease bladder overactivity, and/or to lower the prostatic smooth muscle tonus. Numerous drugs have become available, including α-blockers, 5α-reductase inhibitors (5ARIs), antimuscarinics, phosphodiesterase type 5 inhibitors (PDE5Is), and herbal medicinal products, used alone or in combination, and several others are under evaluation with the results of phase 2/3 studies still pending (Peyronnet et al., 2018). However, to date, all these drugs but 5ARIs have only demonstrated symptomatic effects, with no impact on the natural evolution of the disease (Oelke et al., 2013).

Chronic prostatic inflammation is an old concept (Moore, 1937). Sparse infiltrations with T/B lymphocytes and macrophages are already detected in the epithelial and/or stromal compartments of healthy prostates (De Nunzio et al., 2016). In many instances, the frequency of immune cells drastically increases in hyperplastic prostates, but it is only within the past decade that this feature has been recognized as a risk factor for disease progression. Indeed, a clear association could be established between chronic prostatic inflammation, LUTS severity, and acute urinary retention (Kramer and Marberger, 2006; Mishra et al., 2007; Delongchamps et al., 2008; Nickel et al., 2008; Wang et al., 2008; De Nunzio et al., 2011, 2016; Gandaglia et al., 2013). It has been proposed that inflammatory infiltrates lead to tissue damage and to a chronic process of wound healing that might subsequently promote prostatic enlargement (Gandaglia et al., 2013). The underlying biochemical pathways are highly complex (De Nunzio et al., 2016). These infiltrated immune cells are associated with elevated levels of cytokines/chemokines and their receptors (Kramer et al., 2002; Robert et al., 2011), including IL-2 in epithelium, IL-6 in both stromal and luminal epithelial cells (Royuela et al., 2004), IL-8 in epithelial cells (Giri and Ittmann, 2001), IL-7 and -15 in stromal cells (Mengus et al., 2011), and IL-17 (Steiner et al., 2003). These factors are suspected to act directly on prostatic cells by altering common cellular mechanisms participating in the initiation or the promotion of prostate lesions, e.g., proliferative and anti-apoptotic pathways (Kramer and Marberger, 2006; Wang et al., 2008; Penna et al., 2009; Robert et al., 2009). Based on these clinical, cellular, and molecular evidence, inflammation is emerging as a therapeutic target of interest in the management of BPH patients.

Herbal medicinal products, like antimuscarinics and PDE5Is, do not exert any sexual adverse events (Bschleipfer and Burkart, 2018). As a result, they are the most widely used medication by patients with bothersome LUTS (Lukacs et al., 2013). Herbal products are usually derived from roots, berries, leaves, or fruits of various plants and are provided as extracts containing several chemical compounds (e.g., phytosterols, free fatty acids, flavonoids, triterpenic acids, lignans, and coumarins). The most frequently used herbal drugs for LUTS treatment involve extracts of the fruits of saw palmetto (*Sabal serrulata* synonym to *Serenoa repens*) and the roots of stinging nettle (*Urtica dioica*) (Koch, 2001).

WS^®^ 1541, the active ingredient of Prostagutt forte^®^ is a fixed combination of lipophilic extract from fruits of *S. serrulata* [160 mg WS^®^ 1473; drug–extract ratio 10.0-14.3: 1; extraction solvent: 90% ethanol (m/m)] and an aqueous ethanolic extract from roots of *U. dioica* [120 mg WS^®^ 1031, drug–extract ratio 7.6-12.5: 1; extraction solvent: 60% ethanol (m/m)]. Both constituents of WS^®^ 1541 have a very long tradition as herbal remedies in folk medicine of Europe and the United States. Fruits of saw palmetto (*S. serrulata*) have been used by American Indians to treat a variety of urinary and reproductive system problems (Bennett and Hicklin, 1998). Some of this tradition has come to Europe in the late 19th century where lipophilic extracts from saw palmetto fruits are now widely used for the relief of lower urinary tract symptoms related to benign prostatic hyperplasia (Koch, 2001). Likewise, nettle root has been used in folk medicine as diuretic, against dropsy, for rheumatic disorders, alleviation of gout, and the treatment of prostatic complaints (Frank et al., 1998).

Placebo- (Metzker et al., 1996; Lopatkin et al., 2005, 2007) and reference-controlled trials involving finasteride and tamsulosin (Sökeland and Albrecht, 1997; Sökeland, 2000; Engelmann et al., 2006) have demonstrated the efficacy of WS^®^ 1541 and its non-inferiority to synthetic medications in the treatment of LUTS linked to BPH. These beneficial clinical effects result from the intrinsic pharmacological properties of its two constitutive herbal components that may result in synergistic effects when combined as in WS^®^ 1541 (Koch, 2001). Indeed, lipophilic extracts from saw palmetto fruits have been shown to exert multiple modes of action including antiandrogenic, anti-inflammatory/anti-oedematous, spasmolytic, and antiproliferative effects (Koch, 2001; Geavlete et al., 2011). These activities are mediated through inhibition of 5α-reductase (Niederprum et al., 1994), dual inhibition of cyclooxygenase and 5-lipoxygenase (Breu et al., 1992), inhibition of α1-adrenoceptors (Goepel et al., 1999), as well as inhibition of growth factors (Di Silverio et al., 1998). Likewise, for aqueous–ethanolic stinging nettle root extracts inhibition of aromatase (Koch, 2001), inhibition of leukocyte elastase (Koch et al., 1995), inhibition of membrane Na^+^, K^+^-ATPase activity, which may limit prostate-cell metabolism and growth (Hirano et al., 1994) as well as immunomodulating activity (Koch, 1995), have been reported. The phytochemical complexity of WS^®^ 1451 presumably explains such a functional pleiotropy. However, the actual molecular and cellular targets of WS^®^ 1541 and its two herbal constituents are still poorly characterized in the *in vivo* context of BPH, in part due to the paucity of relevant animal models of the disease (Parisotto and Metzger, 2013).

Experimentally induced systemic hyperprolactinemia (excess of circulating prolactin [PRL]) in rats (Van Coppenolle et al., 2001) and in mice (Wennbo et al., 1997) results in drastic prostate hypertrophy exhibiting features of human BPH such as epithelium hyperplasia and increased stromal cellularity. PRL over-expression in luminal prostate cells using the prostate-specific probasin promoter (Pb–PRL mice) was shown to reproduce these phenotypes without altering androgen serum levels, indicating that prostate hyperplasia in these models was not the results of androgen elevation (Kindblom et al., 2002, 2003). Of note, hypertrophic prostates of Pb–PRL mice display stromal infiltration of lymphocytes and macrophages as typically observed in the human disease (Kindblom et al., 2003; Bernichtein et al., 2015a). Furthermore, functional analyses of Pb–PRL mice revealed that their uroflow pattern was similar to that of BPH patients with LUTS (Lai et al., 2013). Based on these findings, Pb–PRL mice appear to be a good preclinical model of human BPH, and accordingly, it was successfully used to determine the effects of milk diet (Bernichtein et al., 2015b), dietary calcium and vitamin D (Bernichtein et al., 2017), and hexanic lipidosterolic extract of Saw Palmetto *S. repens* (Bernichtein et al., 2015a) on disease progression.

The main objective of the present study was to take advantage of this validated *in vivo* model of BPH to characterize the effects of WS^®^ 1541 on prostate hyperplasia *in vivo*, with particular focus on the inflammatory phenotype. Based on its use in BPH patients, finasteride was used as the reference drug.

## Materials and Methods

### The Pb–PRL Mouse Model

The Pb–PRL mouse is an acknowledged model of inflammatory prostate hyperplasia (Kindblom et al., 2003; Bernichtein et al., 2015a,b). This transgenic model involves local, androgen-regulated (Pb promoter-controlled) overexpression of PRL starting at puberty (4–5 weeks of age) (Kindblom et al., 2003). This leads to over-activation of the STAT5 pathway (Sackmann-Sala et al., 2014) and ultimately to prostate hypertrophy (all prostate lobes affected). Circulating levels of PRL and androgen are normal. Pb–PRL mice have been shown to exhibit several features of human BPH, including tissue hypertrophy, stromal/epithelial hyperplasia, prostate intraepithelial neoplasia (PINs), inflammation, and LUTS (Rouet et al., 2010; Lai et al., 2013; Bernichtein et al., 2015a,b).

### Experimental Design

This study was approved by the local ethics committee (Comité d’Ethique en matière d’Expérimentation Animale Paris Descartes, authorization APAFIS #11744 No. 2017051814533418). Sixty heterozygous C57/Bl6J Pb–PRL transgenic male littermates were generated. One mouse exhibiting signs of skin lesions after fighting was discarded before the study. At the time treatments were started, mice were aged between 21.6 and 25.2 weeks (∼6 months). They were randomly distributed among the various treatment groups, each of which involved 11–12 animals (Table 1). Mice were housed in polycarbonate cages in an environment-controlled room at 22°C on a 12-h dark/light cycle and fed *ad libitum* with standard chow diet (Ref no. 2018, Teklad Global 18% Protein Rodent Diet Harlan, United States; ∼20–22 kcal/day) and water *ad libitum*. They were regularly checked for signs of distress during the course of the study. No adverse effects were noticed.

**Table 1:** Averaged crude values of mouse and prostate weights **(A)** and of immunohistochemical quantifications of epithelial proliferation shown as % of Ki-67+ epithelial cells **(B)** and prostate inflammation shown as number of CD45+ cell foci **(C)** for each treatment group.

	Vehicle	Finasteride	WS^®^ 1541		
mg/kg/day	/	5	300	600	900

**Number of animals/group**	**12**	**12**	**12**	**12**	**11**
**A. Weights**
Mouse (g)	34.21 ± 1.05	33.17 ± 0.76	31.52 ± 0.49	37.25 ± 0.74	34.96 ± 0.83
Half prostate (mg)	61.98 ± 2.27	56.9 ± 3.46	56.7 ± 3.46	55.17 ± 1.76	48.7 ± 1.18
Dorsal lobe (mg)	17.86 ± 1.16	16.51 ± 1.68	16.7 ± 1.54	17.22 ± 0.75	13.79 ± 0.74
Lateral lobe (mg)	16.29 ± 0.48	17.6 ± 1.71	17.33 ± 1.85	14.34 ± 0.66	11.59 ± 0.44
Ventral lobe (mg)	27.83 ± 1.11	22.79 ± 0.97	22.67 ± 0.89	23.62 ± 0.92	23.32 ± 0.97
**B. Ki-67 IHC**
Half prostate	18.2 ±*0.23*	16.7 ±*0.21*	16.4 ±*0.39*	16.8 ±*0.55*	16.4 ±*0.72*
Dorsal lobe	19.7 ±*0.93*	17.8 ±*0.24*	18.8 ±*0.35*	17.7 ±*0.49*	18.2 ±*0.44*
Lateral lobe	12.4 ±*0.77*	11.9 ±*0.68*	11.6 ±*0.24*	13.1 ±*0.62*	12.4 ±*0.88*
Ventral lobe	20.1 ±*0.74*	18.4 ±*0.68*	17.5 ±*0.97*	18.4 ±*0.57*	17.5 ±*1.02*
**C. CD45 IHC**
Half prostate	21.6 ± 1.07	36.5 ± 0.97	22.9 ± 1.04	18.5 ± 1.54	17.7 ± 0.61
Dorsal lobe	4.4 ± 0.20	10.0 ± 0.61	5.9 ± 0.47	5.2 ± 0.54	3.6 ± 0.21
Lateral lobe	6.0 ± 0.25	10.1 ± 0.49	6.2 ± 0.64	4.8 ± 0.54	5.6 ± 0.18
Ventral lobe	11.1 ± 0.53	16.4 ± 0.44	10.8 ± 0.86	8.5 ± 0.82	8.5 ± 0.32

*Normalized data are presented in Figure 2, 3A, 5B–C, respectively.*

Mice were submitted to various doses of WS^®^ 1541 (provided by Willmar Schwabe GmbH & Co. KG), finasteride (Ref. 34202, Sigma), or vehicle only (olive oil, Ref. 01514, Sigma). Doses were chosen based on efficacy reported in former studies for finasteride (Van Coppenolle et al., 2000) and on data from a previous investigation with WS^®^ 1541 in a sulpiride-induced rat BPH model (Kraft et al., 2016).

– Group 1: Vehicle (oil, 5 ml/kg)
– Group 2: WS^®^ 1541 300 mg/kg/day dissolved in 5 ml/kg vehicle
– Group 3: WS^®^ 1541 600 mg/kg/day dissolved in 5 ml/kg vehicle
– Group 4: WS^®^ 1541 900 mg/kg/day dissolved in 5 ml/kg vehicle
– Group 5: finasteride 5 mg/kg/day dissolved in 5 ml/kg vehicle

For the study, a single batch of WS^®^ 1541 (Material No. 0891) was used. This corresponds to the finished medicinal product containing 35% WS^®^ 1473, 27% WS^®^ 1031, and 38% excipients. Based on allometric scaling the low dose of WS^®^ 1541 (300 mg/kg) is nearly equivalent to the therapeutic dose in men (560 mg/day). Compounds were administered daily (in the morning) for 28 consecutive days by oral gavage as previously described (Bernichtein et al., 2015a). Mice were weighed once a week. At the end of the treatments, animals were sacrificed by cervical dislocation.

To isolate the prostate, dissection of the urinary tract was performed and left lobes were separately dissected, weighed, then rapidly snap frozen while the right-sided half prostate was fixed in paraformaldehyde (PFA) without being further dissected, so that tissue organization was preserved for histological analysis. We analyzed the three most commonly studied prostate lobes (ventral, lateral, dorsal).

### Histology and Immunohistochemistry

Tissues were fixed in 4% PFA in PBS overnight then in 50% ethanol before being processed for histological studies. Serial sections (4 μm thickness; 1 section per slide) were performed for histological and immunohistochemical (IHC) analyses. For histology, sections were stained with classical hematoxylin–eosin (H&E). Fields were selected following systematic random sampling scheme. For IHC studies, paraffin-embedded/PFA-fixed sections were deparaffinized in a xylene substitute (Neo-Clear^®^) and rehydrated in graded ethanol. Endogenous peroxidase activity was blocked by incubating slides in 3% H_2_O_2_ for 10 min at room temperature, and non-specific binding of immunoglobulins was minimized by pre-incubation with 2% normal horse serum in PBS for 30 min at room temperature. Sections were boiled for 30 min in citric acid (pH 6.0) for antigen retrieval. References and conditions of use of the various primary antibodies are listed in Table 2. The avidin biotin immunoperoxidase system was used to visualize primary antigen–antibody complexes (Vectastain Elite ABC Kit; Vector Laboratories, Burlingame, CA, United States) using 3,3’-diaminobenzidine as chromogen (SK-4100; Vector Laboratories, Burlingame, CA, United States). Slides were then counterstained with hematoxylin.

**Table 2:** Antibody references and conditions of use.

	Species	Ref/clone	Company	Dilution	Incubation	Purpose
CD45	Rat monoclonal	sc-53665/30-F11	Santa Cruz	1/150	O/N, 4°C	Inflammation
Ki-67	Rabbit monoclonal	RM9106/SP6	Thermo Scientific	1/300	O/N, 4°C	Proliferation
P-Stat5	Rabbit monoclonal	9359/C11C5	Cell Signaling	1/300	O/N, 4°C	Transgene activity

### Prostate Histopathology

Histopathological diagnosis of prostate sections from all mice was performed in blind by an independent veterinary pathologist (ER-G) trained for mouse prostate analysis (Bernichtein et al., 2017) following the recommendations of the Mouse Models of Human Cancer Consortium Prostate Pathology Committee and the reference classification of PIN lesions in genetically modified animals (Park et al., 2002; Shappell et al., 2004). This qualitative analysis was complemented by quantitative IHC analyses as described below.

### Image Acquisition and Histology Quantifications

Prostate tissue sections (H&E or IHC) were digitally scanned using a NanoZoomer-2.0 RT scanner (Hamamatsu, Photonics, France) coupled to NDP.view2 software analysis beta version U12388-01 (Hamamatsu, Photonics, France). The latter was used for quantification of STAT5-positive nuclei in epithelial cells (reflecting transgene activity) and CD45-positive cell foci (reflecting inflammation) as previously described (Bernichtein et al., 2015a,b).

**FIGURE 6:**
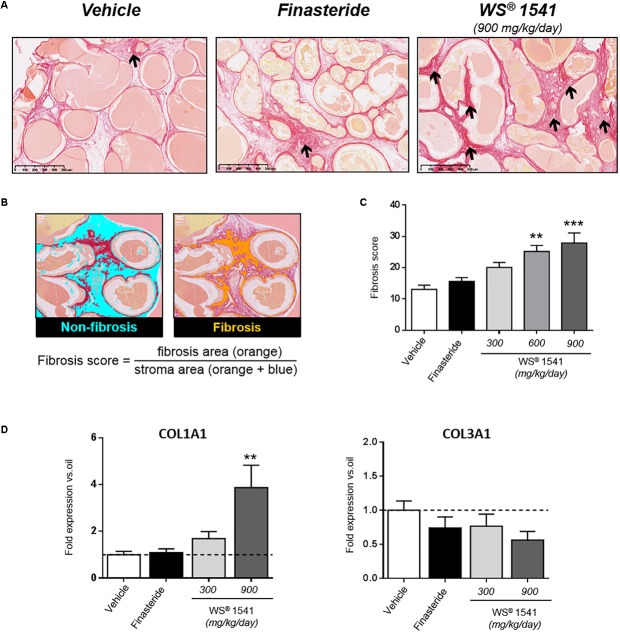
Prostate fibrosis. **(A)** Representative images of picrosirius red staining showing stromal fibrosis in the ventral prostate. Arrows point to dense, isolated fibrotic stained foci in the vehicle- and finasteride-treated groups whereas staining is evenly distributed in the WS^®^ 1541-treated groups, especially at the highest dose shown here. **(B)** Illustration of fibrosis score calculation by image analysis using Calopix^®^ software (see the section “Materials and Methods” for explanations). **(C)** Fibrosis score in the various treatment groups. Symbol: ^∗^ vs. vehicle group, ANOVA, followed by Tukey’s, *n* = 11–12 per group. **(D)** Expression level of profibrotic genes COL1A1 and COL3A1 was assessed by RT-qPCR in ventral lobe. Results are expressed as fold expression vs. vehicle group.

For the quantification of fibrosis, picrosirius red staining was performed for all animals from each treatment group as described (Bernichtein et al., 2015a). Next we used Calopix software^1^ to establish the fibrosis score as the ratio of fibrosis and stroma areas as illustrated in Figure 6.

### Quantitative RT-PCR

Total RNA was isolated from separate prostate lobes using the NucleoSpin^®^ RNA XS (Macherey Nagel, Hoerd, France) according to manufacturer’s instructions. RNA integrity was assessed on Agilent BioAnalyzer (all RNAs scored 7–10). RNA (250 ng) was reverse transcribed using SuperScript^TM^II Reverse transcriptase with the SuperScript^TM^II First-Strand Synthesis System for RT-PCR Kit (Invitrogen, Carlsbad, CA, United States). The cDNAs were then subjected to real-time PCR amplification using gene-specific primers (0.5 μM final concentration) purchased from IDT DNA (Integrated DNA Technologies, BVBA, Leuven, Belgium; HPLC purification; referred to as Mm.PT in Table 3) or Eurogentec (Liège, Belgium; Oligold quality, sequence given in Table 3). Peptidyl prolyl isomerase A (PPIA) that encodes cyclophilin A was used as the housekeeping gene in each reaction. Real-time PCR was performed using a Qtower 2.0 (Analytik Jena, Germany). The qPCR reaction contained 2 μl cDNA sample (12.5 ng) and 8 μl mastermix with 1× GoTaq^®^ qPCR Master Mix (Promega) and 0.5 μM primer. The Qtower 2.0 Instrument was used with the following program: Enzyme activation: 95°C for 2 min; amplification (40 cycles): 95°C for 15 s, 60°C for 60 s. Results were generated with the Qtower 2.0 software and were analyzed by the comparative cycle threshold method and presented as fold change in gene expression relative to internal calibrators as mentioned in figures. Experiments were performed in duplicate and the results are expressed as means ± *SD*.

**Table 3:** Sequences of primers used for quantitative RT–PCR.

Gene	Name	5′–3′ sequence
*Cyclophilin*	Cyclophylin A-R	TTGCTGGTCTTGCCATTCCT
	Cyclophilin A-F	CAGGTCCTGGCATCTTGTCC
*Ki-67*	Mouse Ki67-F	AAAGGCGAAGTGGAGCTTCT
	Mouse Ki67-R	TTTCGCAACTTTCGTTTGTG
*Bcl-2*	B-cell lymphoma 2-R	AGTACCTGAACCGGCATCTG
	B-cell lymphoma 2-F	CATGCTGGGGCCATATAGTT
*IL-1β*	Interleukin-1beta	Mm.PT.49a.17212823
*IL-2*	Interleukin-2-F	TGAGCAGGATGGAGAATTACAGG
	Interleukin-2-R	GTCCAAGTTCATCTTCTAGGCAC
*IL-6*	Interleukin-6	Mm.PT.58.13354106
*IL-15*	Interleukin-15	Mm.PT.58.7522730
*IL-17a*	Interleukin-17A	Mm.PT.58.6531092
*CXCL1*	Chemokine ligand 1	Mm.PT.58.42076891
*CXCL6*	Chemokine ligand 6	Mm.PT.58.11287813
*CXCL10*	IFN-γ-inducible protein 10	Mm.PT.58.43575827
*CCL2*	Chemokine ligand 2	Mm.PT.58.42151692
*CCL4*	Chemokine ligand 4	Mm.PT.58.5219433
*CCL7*	Chemokine ligand 7	Mm.PT.58.17719534
*CCL8*	Mouse CCL8-F	CAGTGCTTCTTTGCCTGCTG
	Mouse CCL8-R	ACATGAAAGCAGCAGGTGAC
*CCR7*	Chemokine receptor 7	Mm.PT.58.31257202
*CXCR4*	Chemokine receptor 4	Mm.PT.58.41597935
*CD40LG*	CD40 ligand	Mm.PT.58.6864937
*CTLA4*	Cytotoxic T-lymphocyte-associated protein 4	Mm.PT.58.30832695
*COX2*	Cyclo-oxygenase-F	GGTCATTGGTGGAGAGGTGTAT
	Cyclo-oxygenase-R	TGAGTCTGCTGGTTTGGAATAG
*iNOS*	Nitric oxide synthase 2-F	GTTCTCAGCCCAACAATACAAGA
	Nitric oxide synthase 2-R	GTGGACGGGTCGATGTCAC
*TNFα*	Tumor necrosis factor-F	TGTGAAGGGAATGGGTGTTC
	Tumor necrosis factor-R	TGAGACAGAGGCAACCTGAC
*FGF2*	Fibroblast growth factor 2	Mm.PT.56a.5129235
*TGFβ*	Transforming growth factor, beta 1-F	CTCCCGTGGCTTCTAGTGC
	Transforming growth factor, beta 1-R	GCCTTAGTTTGGACAGGATCTG
*Egr1*	Early growth response 1-F	TCGGCTCCTTTCCTCACTCA
	Early growth response 1-R	CTCATAGGGTTGTTCGCTCGG
*EGF*	Epidermal growth factor-F	AGCATCTCTCGGATTGACCCA
	Epidermal growth factor-R	CCTGTCCCGTTAAGGAAAACTCT
*COL1A1*	Collagen 1A1	Mm.PT.58.7562513
*COL3A1*	Collagen 3A1	Mm.PT.58.41847308

### Statistics

All quantitative data are expressed as mean ±*SD* and all comparisons were made with 11–12 animals per group using one-way ANOVA followed by Tukey’s comparison test (GraphPad Software, version 5.00 for Windows, San Diego, CA, United States, www.graphpad.com). *P*-values between treatment groups (finasteride and WS^®^ 1541) and vehicle are represented on figures as follows: (^∗^) when *p* < 0.05, (^∗∗^) when *p* < 0.01, and (^∗∗∗^) when *p* < 0.001.

## Results

### Treatment Tolerance and Validation

**FIGURE 1:**
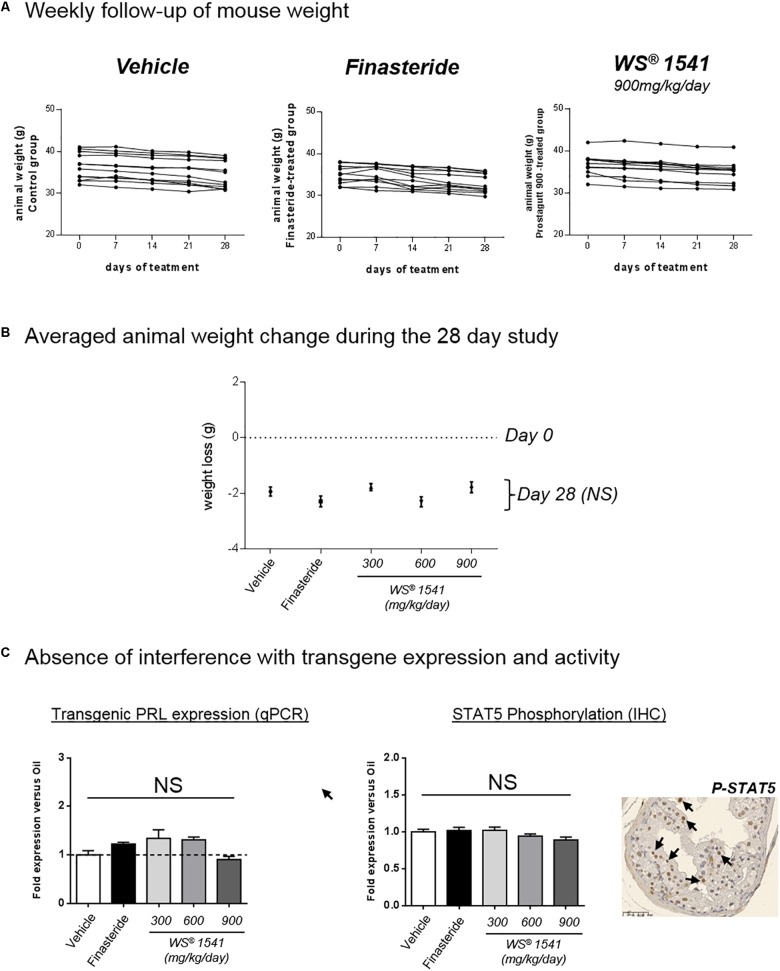
Treatment tolerance and validation. **(A)** The weight of each animal (g) was measured at 0, 7, 14, 21, and 28 days of treatment. Each curve represents one single animal, *n* = 11–12 animals per group. **(B)** Averaged animal weight change in each treatment group between day 0 (dotted line) and sacrifice at day 28 of treatment (black dots). No significant (NS) difference was observed for any treatment group compared to vehicle (Mann–Whitney test). **(C)** Evaluation of the PRL/STAT5 pathway in all groups. *Left*: the mRNA expression of the rPRL transgene was determined by qPCR in the three lobes. Results of dorsal lobe are shown as fold expression vs. vehicle group. *Right*: the level of STAT5 activation in the various lobes and various treatment groups was analyzed by immunohistochemistry using anti-phosphorylated STAT5 antibodies. One typical image of dorsal lobe is shown (vehicle group), in which some nuclei positive for pSTAT5 are identified by black arrows. The ratio of STAT5-positive *versus* total nuclei in the epithelium was quantified as described in the section “Materials and Methods,” and the results of dorsal lobe are expressed as fold expression vs. vehicle group. ANOVA was used as statistical test followed by Tukey’s (*n* = 11–12 animals per group).

Treatments by oral gavage lasted for 28 consecutive days. As earlier reported (Bernichtein et al., 2015a) mice tolerated well this mode of administration, with no obvious sign of adverse effects. Accordingly, weekly measurement of body weight did not show marked alteration during the treatment in control, finasteride, and WS^®^ 1541 groups (Figure 1A). At sacrifice, a slight decrease in averaged body weight (∼2 g/mouse, not significantly different between groups; Figure 1B) was noticed in all groups including vehicle, suggesting it was due to the stress of daily handling and gavage and not to any specific effect of the compounds. Taken together, these results confirmed the good tolerance of the oral-route treatments and established the administration of up to 900 mg/kg/day of WS^®^ 1541 for consecutive 28 days as not toxic for experimental mice.

In order to avoid misinterpretation of the data reported below, we ensure that the treatments did not interfere with transgene expression or activity. To that end we evaluated the status of the PRL-STAT5 signaling pathway which drives prostate hyperplasia in Pb-PRL mice (Sackmann-Sala et al., 2014). The levels of rat PRL transgene mRNA (as measured by qPCR) and of STAT5 tyrosine phosphorylation (i.e., activation) in the epithelium (as measured by IHC) were not altered by WS^®^ 1541 or finasteride treatment in any of the three lobes (dorsal lobe shown as an example in Figure 1C). Consequently, any difference between vehicle and compound-treated groups should be attributable to intrinsic actions of the compound(s) rather than to interference with transgene activity.

### WS^®^ 1541 Treatment Reduces Prostate Tissue Weight

**FIGURE 2:**
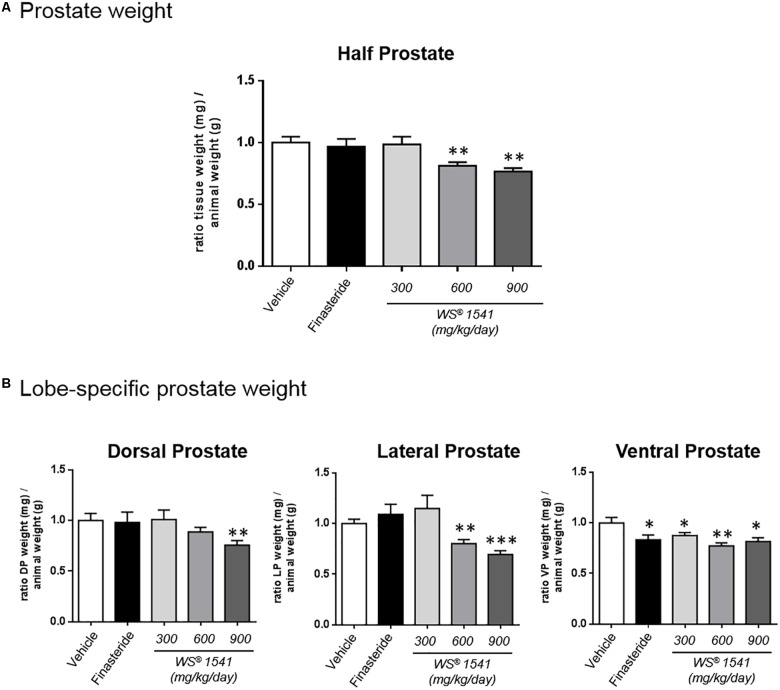
Prostate weights. **(A)** Ratio between tissue weight of the half prostate (sum of the three lobes, mg) and the corresponding animal weight (g). Data are normalized to the control group (vehicle). **(B)** Lobe-specific effect of the various treatments as in panel **A**. Data are normalized to the vehicle group. Symbol: ^∗^ vs. vehicle group, ANOVA followed by Tukey’s.

At 6 months of age Pb-PRL mice exhibit highly hypertrophic prostates compared to age-matched WT littermates (Rouet et al., 2010; Bernichtein et al., 2015a). In this study, macroscopic examination of the prostates at dissection did not reveal obvious alteration of lobe size or morphology between treatment groups. The weights of half prostates (Figure 2A) and of each individual lobe (Figure 2B) were measured then normalized to the corresponding mouse weight (see Table 1A for averaged crude values). As earlier observed (Bernichtein et al., 2015a) the ventral prostate was the most responsive as all treatments significantly decreased the weight of this lobe. The lateral prostate was responsive to the two highest doses of WS^®^ 1541, and dorsal prostate to the highest dose only (Figure 2B). When the prostate was taken as a whole (three lobes combined), the two highest doses of WS^®^ 1541 (600 and 900 mg/kg/day) significantly decreased prostate weight compared to vehicle (Figure 2A); the lowest dose of WS^®^ 1541 (300 mg/kg/day) and finasteride had no effect.

Together, these data indicate that 28 days of daily treatment with 600 and 900 mg/kg/day of WS^®^ 1541 significantly reduced prostate weight of Pb–PRL mice, with the highest dose acting on the three lobes.

### WS^®^ 1541 Treatment Reduces Prostate Cell Proliferation

**FIGURE 3:**
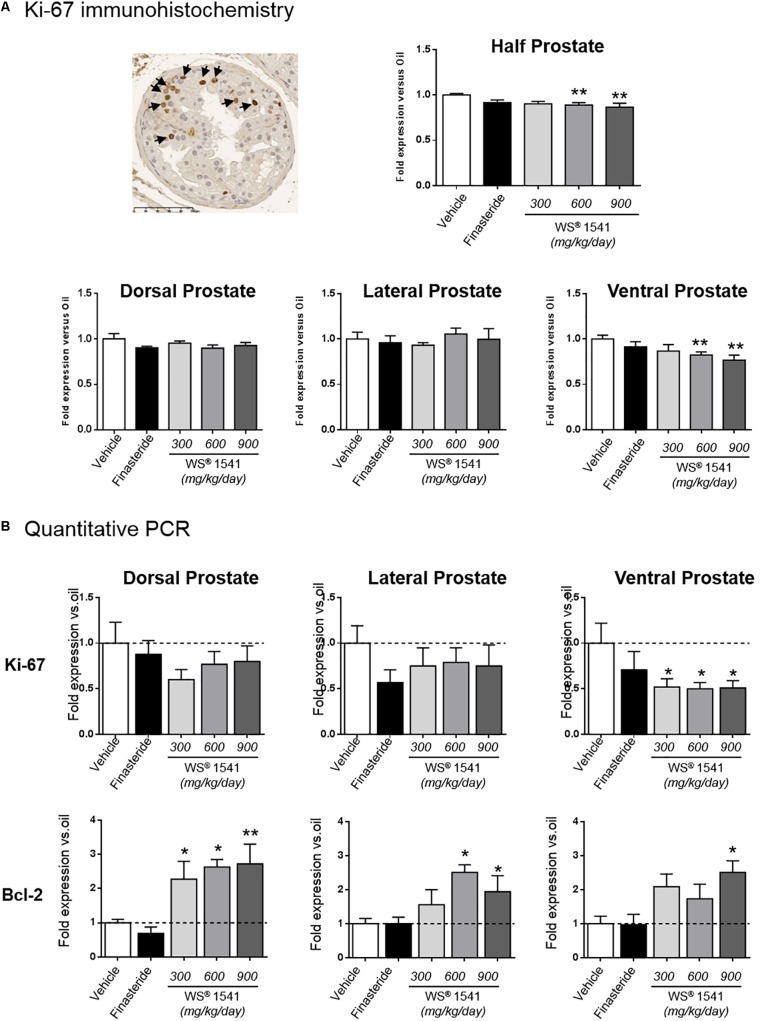
Prostate cell proliferation. **(A)** The epithelial cell proliferation index was determined by Ki-67 immunohistochemistry (one typical image is shown with some positive nuclei identified by arrows). The data obtained for half prostates (top) or each individual lobe (bottom) are shown for each treatment relative to the vehicle group. **(B)** Ki-67 and Bcl-2 expression as determined by qPCR is represented for each individual prostate lobe and is normalized to the vehicle group. Symbol: ^∗^ vs. vehicle group (*n* = 11–12 per group), ANOVA, followed by Tukey’s.

The proliferation index of epithelial cells was determined by Ki-67 IHC as earlier reported (Bernichtein et al., 2015a,b, 2017). As shown in Figure 3A (top panel), the two highest doses of WS^®^ 1541 significantly reduced the epithelial proliferation index in the prostate tissue compared to the vehicle condition (see Table 1B for averaged crude values). This was mainly due to an effect on the ventral prostate, since only a trend was observed in the dorsal lobe, and no effect in the lateral lobe (Figure 3A, bottom panels). Of note, finasteride had no significant effect in any condition.

The measurement of Ki-67 level by qPCR provides an idea of global cell proliferation in the entire tissue including the epithelium and the stroma (fibroblasts, inflammatory cells, etc.). As shown in Figure 3B, WS^®^ 1541 induced a significant reduction of cell proliferation in the ventral prostate, while a trend was observed in the two other lobes. Only a mild trend was observed for finasteride in all lobes.

Bcl-2 is primarily known as a key regulator of apoptosis (Hockenbery et al., 1990). Beyond its anti-apoptotic properties, it has also been recognized as an anti-proliferative factor (Bonnefoy-Berard et al., 2004). A recent study showed that Bcl-2 expression was down-regulated in human BPH specimens, in agreement with tissue growth (Li et al., 2018). As shown in Figure 3B, Bcl-2 was upregulated by WS^®^ 1541 treatment in the three lobes, with dorsal lobe showing the highest sensitivity. Finasteride had no effect on this parameter, in agreement with what was reported in human BPH (Li et al., 2018).

In summary, these results show that WS^®^ 1541 reduced various hallmarks of cell proliferation with lobe-specific sensitivity and/or dose-dependent efficacy, as observed for prostate tissue weight (Figure 2A). No significant effect was observed for finasteride treatment.

### WS^®^ 1541 Treatment Does Not Alter Prostate Epithelium Histology

**FIGURE 4:**
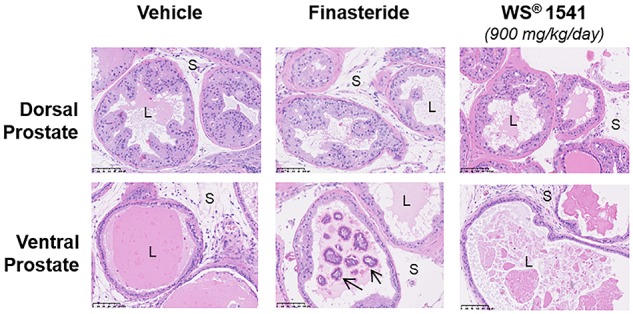
Prostate histopathology. Representative images of dorsal and ventral prostate histology in the various treatment groups (magnification 20×). For WS^®^ 1541 only the highest dose is shown as an example. Intraluminal mineralizations (identified by arrows) were frequently observed in the finasteride group. Symbols: S, stroma; L, lumen.

Careful histopathological analysis of the five treatment groups was performed in blind by a veterinary pathologist (ER-G) trained to mouse prostate analysis (Bernichtein et al., 2017). Global histological observation of half prostates at low magnification (1.5×) allows to visualize the architecture of dorsal, lateral, and ventral prostate lobes around the urethra, as it stands *in situ*. No major alteration could be noticed in either treatment groups (not shown), suggesting that neither WS^®^ 1541 nor finasteride treatment affect the gross architecture of the prostate tissue compared to vehicle-treated animals. The lobe-specific epithelial cell phenotypes as they can be observed at higher magnification (20–40×) have been earlier reported (Bernichtein et al., 2015a,b); there was no relevant histopathological differences among the various groups of the present study (Figure 4). The sole noticeable phenotype was, in the finasteride group only, the high frequency (5 mice out of 11) of intraluminal mineralization possibly corresponding to calcified *Corpora amylacea*, also reported as “prostate stones” (Köseoglu, 2012; Figure 4).

### WS^®^ 1541 Treatment ReducesProstatic Inflammation

Moderate chronic inflammation of the prostatic stroma has been well described in the Pb–PRL model, with stromal mononuclear infiltrates mainly composed by lymphocytes and macrophages (Kindblom et al., 2003). As earlier reported the stromal cellularity gradually increased from dorsal-to-ventral lobes (Bernichtein et al., 2015a).

**FIGURE 5:**
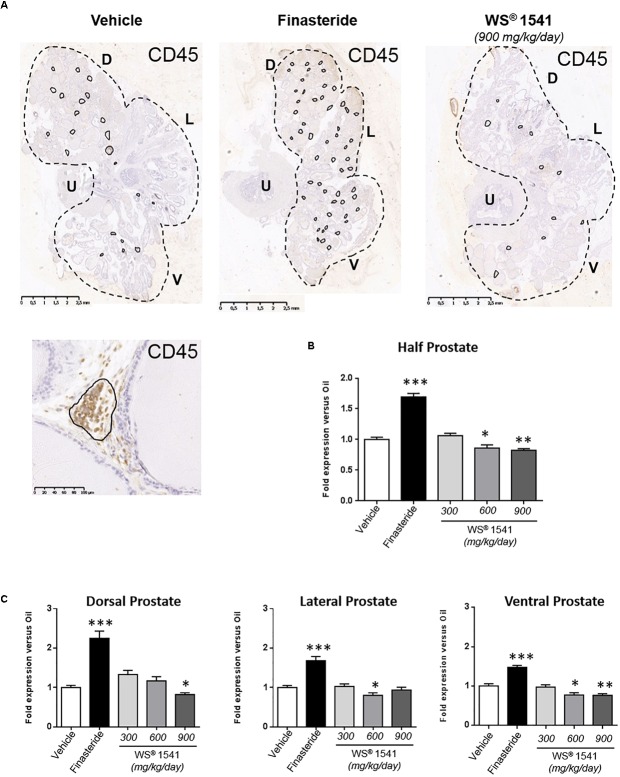
Prostate inflammatory status. **(A)** Representative illustrations of CD45 immunostaining of half prostate sections from the various groups (only the highest dose is shown for WS^®^ 1541). CD45-positive cell foci are circled in black, and one typical foci is shown at high magnification. The number of CD45-positive cell clusters was quantified for half prostate **(B)** and for each individual lobe **(C)** by image analysis as described in the section “Materials and Methods.” The results are normalized to the vehicle condition (see Table 1C for averaged crude values). Symbol: ^∗^ vs. vehicle group, ANOVA, followed by Tukey’s. D, dorsal prostate; L, lateral prostate; V, ventral prostate; U, urethra.

CD45 (leukocyte common antigen) IHC is commonly used to monitor tissue inflammation (Bernichtein et al., 2015a). Figure 5A illustrates the number of CD45+ cell foci in half prostate sections from various treatment groups, and Figure 5B (half-prostate) and 5C (lobe-specific) quantify this parameter as fold-change relative to the vehicle group (see Table 1C for averaged crude values). Finasteride globally increased the prostatic inflammatory status as the number of CD45+ cell foci increased in all lobes. In addition, the size of these foci was also increased in two animals (not shown). Conversely, WS^®^ 1541 treatment reduced prostate inflammation in a dose-dependent manner, being more efficient at 900 than 600 mg/kg/day; the lowest dose had no significant effect. Again, the ventral lobe was more responsive than the two other lobes (Figure 5C).

Fibrosis, i.e., deposition of extracellular collagen by activated fibroblasts, can be a sequelae of healing inflammation (Shappell et al., 2004). This phenotype can be visualized (Figure 6A) and quantified (Figure 6B) using picrosirius red staining. In the Pb–PRL model, mild fibrosis is observed mainly in the ventral prostate, in good agreement with the higher stromal cellularity of this lobe. There was a dose-related escalation of fibrosis in ventral prostate after 28 days of treatment with WS^®^ 1541 that was significant at the highest doses both at the histological level (Figure 6C) and at the molecular level as reflected by the upregulation of collagen 1 A1 (but not collagen 3 A1) (Figure 6D). In fact, this fibrosis phenotype was the mirror image of inflammation (Figure 5B), suggesting it reflects scarring fibrosis that developed as a consequence of healing inflammation. Consistent with this hypothesis, the persistent inflammatory status observed in the finasteride group was not linked to increased fibrotic phenotype (Figure 6C).

### WS^®^ 1541 Treatment Reduces the Pro-inflammatory Molecular Profile of Pb–PRL Prostates

Using an expression array of pro-inflammatory cytokines/chemokines, we earlier showed that many pro-inflammatory genes were deregulated in Pb–PRL *versus* WT prostates, a phenotype that aggravated with age (Bernichtein et al., 2015a). Based on that study and on other recent findings from some of us (Kraft et al., 2016), we selected a panel of 23 genes encoding pro-inflammatory cytokines, chemokines, receptors, enzymes, and growth factors (Table 4) and we analyzed their expression with the aim to provide molecular relevance to reduction of immune cell infiltrates reported in Figure 5.

**Table 4:** List of screened pro-inflammatory genes.

Short name	Name	Class
IL-1β	Interleukin-1beta	Pro-inflammatory cytokine
IL-2	Interleukin-2	Pro-inflammatory cytokine
IL-6	Interleukin-6	Pro-inflammatory cytokine
IL-15	Interleukin-15	Pro-inflammatory cytokine
IL-17a	Interleukin-17a	Pro-inflammatory cytokine
CD40lg	CD40 ligand (T-cell surface protein)	Pro-inflammatory cytokine
TNFα	Tumor necrosis factor alpha	Pro-inflammatory cytokine
CCL2	C–C motif chemokine ligand 2	Chemokine
CCL4	C–C motif chemokine ligand 4	Chemokine
CCL7	C–C motif chemokine ligand 7	Chemokine
CCL8	C–C motif chemokine ligand 8	Chemokine
CXCL1^∗^	C–X–C motif chemokine ligand 1	Chemokine
CXCL6	C–X–C motif chemokine ligand 6	Chemokine
CXCL10	C–X–C motif chemokine ligand 10	Chemokine
CCR7	C–C motif chemokine receptor 7	Chemokine receptor
CXCR4	C–X–C motif chemokine receptor 4	Chemokine receptor
CTL4A	Cytotoxic T-lymphocyte-associated antigen 4	Immune cell receptor
COX-2	Cyclooxygenase 2 (prostaglandin–endoperoxide synthase)	Pro-inflammatory enzyme
iNOS	Inducible nitric oxide synthase	Pro-inflammatory enzyme
FGF2	Fibroblast growth factor 2	Growth factor
EGF	Epidermal growth factor	Growth factor
TGFβ	Transforming growth factor beta	Growth factor
Egr1	Early growth response 1	Transcription factor

*^∗^Homologous to human IL-8 (Hol et al., 2010).*

**FIGURE 7:**
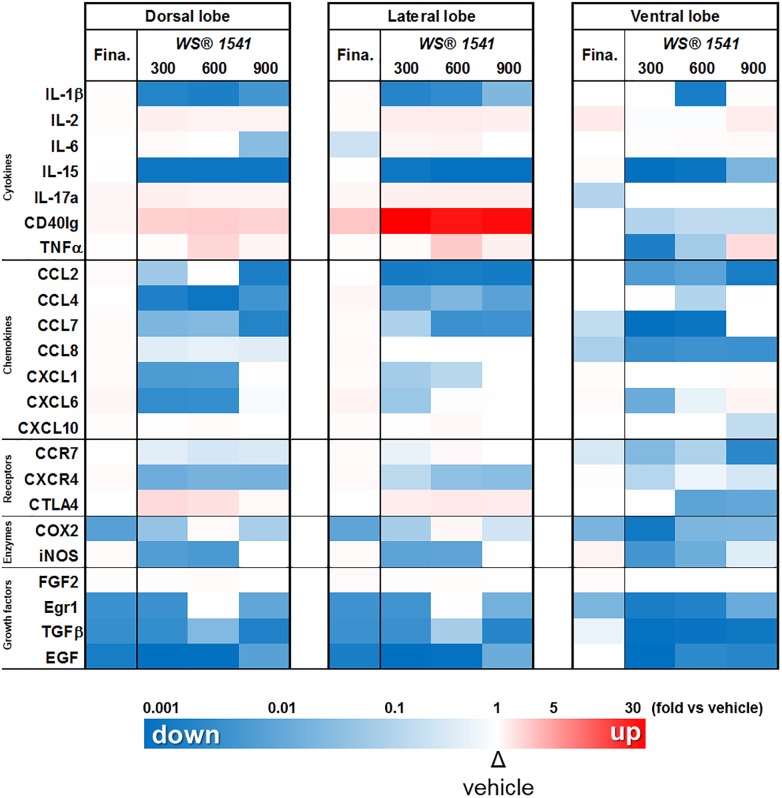
WS^®^ 1541 treatment reduces the pro-inflammatory molecular profile of Pb–PRL prostates. Expression of 23 selected pro-inflammatory factors in the dorsal, lateral, and ventral prostate lobes from the various treatment groups was assessed by RT-qPCR (*n* = 11–12). Results are presented as a heatmap with respect to the vehicle group [the highest induction (red) corresponds to 32-fold, the lowest (blue) to 0.003-fold]. Fina, finasteride.

The relative level of expression of the 23 genes of interest in the three lobes following WS^®^ 1541 (three doses) or finasteride treatment, normalized to the vehicle group for each lobe, is displayed in Figure 7 as a color-coded heatmap (upregulation in red, downregulation in blue, unchanged in white, with color intensity proportional to the fold-changes). This representation nicely illustrates that: (i) WS^®^ 1541 downregulated most of the genes tested, especially in the ventral lobe (virtually no red boxes), (ii) the lower dose of WS^®^ 1541 (300 mg/kg/day) was in many instances as efficient as the highest dose to downregulate gene expression (e.g., EGF and IL-15), and (iii) WS^®^ 1541 was much more efficient than finasteride to downregulate pro-inflammatory target genes (much less blue boxes in the finasteride columns).

**FIGURE 8:**
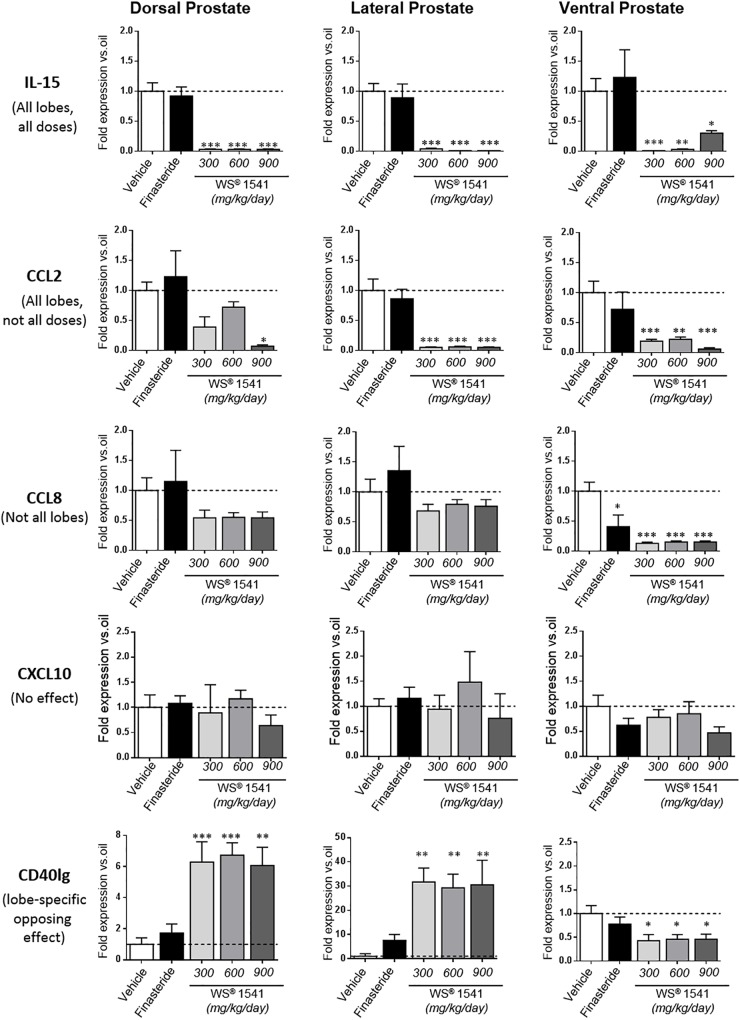
Differential regulation of pro-inflammatory cytokines/chemokines by WS^®^ 1541. Typical examples of gene regulation by WS^®^ 1541: genes that are (i) significantly downregulated in all lobes and at all doses (IL-15), (ii) significantly downregulated in all lobes but not at all doses (CCL2), (iii) significantly downregulated in some but not all lobes (CCL8), (iv) unaltered (CXCL10), and (v) regulated in opposite ways in different lobes (CD40lg). See text for the section “Discussion.” Statistical analysis was performed by ANOVA, followed by Tukey’s.

Closer analysis of the expression data showed five types of responses to WS^®^ 1541; one example of each is illustrated in Figure 8. Three genes (IL-15, EGF, and TGFβ) were significantly downregulated in all lobes and at all doses of WS^®^ 1541. Four genes (CCL2, CCL4, CCL7, and CXCR4) were also significantly decreased in all lobes albeit not all doses of WS^®^ 1541 achieved statistical significance. Eight genes exhibited significant reduction in some lobe(s) and no effect in other(s) (ventral: CCL8, CCR7, COX-2, and Egr1; dorsal/lateral: iNOS and IL-1β; dorsal: CXCL6 and CXCL1). The global picture for the above-cited 15 genes was a reduction of expression by WS^®^ 1541. Three genes were not altered by WS^®^ 1541 treatment (CXCL10, IL-6, FGF2). Regarding the five remaining genes, lobe-dependent opposing effects (up in dorsal/lateral, down in ventral: CD40lg, CTLA4, IL-17a) and/or dose-dependent increase (IL-2, TNFα) were observed. The few genes that were significantly downregulated by finasteride included three growth-related factors (EGF, TGFβ, Egr1) and the enzyme COX-2. With rare exceptions (mild reduction of CCL7/CCL8/IL-17a in ventral lobe) finasteride failed to alter cytokines/chemokines and receptor expression.

## Discussion

Herbal extracts are commonly used worldwide to treat LUTS in patients with BPH. The benefit of using WS^®^ 1541 has been assessed in various clinical studies, including a placebo-controlled trial showing superiority in LUTS improvement after 24 weeks of treatment (Metzker et al., 1996; Sökeland and Albrecht, 1997; Sökeland, 2000; Lopatkin et al., 2005, 2007; Engelmann et al., 2006). To investigate further the mechanisms of action of this herbal drug in the *in vivo* context of BPH, we used the Pb–PRL transgenic mouse model of prostate hypertrophy. Compared to other rodent models of BPH (Wennbo et al., 1997; Van Coppenolle et al., 2001; Nicholson et al., 2012), Pb–PRL mice do not exhibit elevated serum levels of androgens (Kindblom et al., 2003), which better mimics the context of patients developing BPH. This model was recently used to highlight the anti-inflammatory properties of lipidosterolic extract of *S. repens* (Bernichtein et al., 2015a). Following a similar strategy, we here demonstrated that WS^®^ 1541 was able to reduce both prostate tissue growth and inflammation. Importantly, at the doses tested, it was more efficient than finasteride to downregulate these two hallmarks of BPH progression.

### WS^®^ 1541 Reduces Prostate Growth

WS^®^ 1541 involves two herbal extracts: *S. serrulata* (WS^®^ 1473) and *U. dioica* (WS^®^ 1031). The former was shown to exert mild anti-androgenic activity through partial inhibition of 5α-reductase (Niederprum et al., 1994). This effect common to various Saw palmetto fruit extracts was nevertheless drastically lower (<5,000-fold) compared to finasteride, the archetype synthetic 5ARI (Rhodes et al., 1993). This suggests that the therapeutic efficiency of WS^®^ 1541 in BPH patients primarily rely on intrinsic properties other than androgen pathway inhibition. We here show that the two highest doses of WS^®^ 1541 reduced both prostate weight and epithelial cell proliferation. Beyond androgens, prostatic growth is mediated by various growth factors which can originate from epithelial and/or stromal cells e.g., epidermal growth factor (EGF), transforming growth factor beta (TGFβ), or fibroblast growth factors (FGFs) (Allkanjari and Vitalone, 2015). WS^®^ 1541 strongly (*p* < 0.001) downregulated EGF, TGFβ, and the mitogenesis-related *Egr1* transcriptional regulator in the three prostate lobes (Figure 7). It is tempting to speculate that downregulation of these gene products contributed to the reduction of prostate hypertrophy induced by WS^®^ 1541 in the Pb–PRL model. However, their expression was downregulated to similar extent by finasteride (Figure 7), which otherwise failed to alter prostate weight (Figure 2A) and cell proliferation (Figure 3). This suggests either that these growth-related gene products may not be major actors of prostatic growth in the Pb–PRL model, or that the anti-growth effect resulting from their downregulation may depend on other factors differently regulated by WS^®^ 1541 and finasteride. As discussed below, the role of inflammation can be speculated.

### WS^®^ 1541 Reduces Several Hallmarks of Prostatic Inflammation

The anti-inflammatory properties of WS^®^ 1541 have been suggested in a couple of former studies (Koch et al., 1995; Koch, 2001; Kraft et al., 2016). In the present study, the quantitative assessment of both infiltrating immune cells (CD45+) and several selected pro-inflammatory factors highlighted the anti-inflammatory properties of WS^®^ 1541 on the hyperplastic prostates of Pb–PRL mice. Among the pro-inflammatory cytokines/chemokines and receptors that we investigated in this study, we identified IL-1β, IL-15, CCL2, CCL4, CCL7, CXCL1, CXCL6, CXCR4, and CCR7 as the preferential targets of WS^®^ 1541 as all were downregulated in ≥2 lobes and/or at ≥2 doses (Figure 7). IL-1β, IL-15, and CCL2 are secreted by stromal BPH cells and their production is increased when BPH cells are co-cultured with activated CD4+ T cells (Vignozzi et al., 2012a), suggesting they may contribute to a self-sustained pro-inflammatory vicious circle. CXCL1, CXCL6 (Begley et al., 2008), and CXCL12, the CXCR4 ligand (Begley et al., 2005), are secreted by stromal prostate fibroblasts from aging prostates and were shown to promote low-level increase in proliferation of primary prostate epithelial cells at sub-nanomolar concentration. The down-regulation of these ligands and receptor is therefore anticipated to contribute to the beneficial effects of WS^®^ 1541 in the context of BPH.

Cyclooxygenase-2 (COX-2) and inducible nitric oxide (NO)-synthase (iNOS) are two key enzymes in the prostaglandin and NO-synthesis pathways, respectively. In inflammatory cells that infiltrate the prostate, iNOS is the principal factor activating reactive nitrogens that can subsequently damage cells (Baltaci et al., 2001). Furthermore, NO enhances COX activity, leading to the production of prostaglandins known as important regulators of prostate cell proliferation (Sciarra et al., 2007). The expression of iNOS has been shown to be upregulated in patients with BPH (Gradini et al., 1999), as are prostaglandin levels (Robert et al., 2009) and COX activity in proliferative inflammatory prostate lesions (Sciarra et al., 2007). While finasteride reduced only COX-2, WS^®^ 1541 downregulated both COX-2 and iNOS. Interestingly, the lowest dose of WS^®^ 1541 was the most efficient (Figure 7). This supports a stronger effect of the herbal drug on these two connected pathways, which may contribute to the anti-growth effects of WS^®^ 1541.

Finally, two genes of interest (CD40lg and CTLA4) showed paradoxical response to WS^®^ 1541 as they were upregulated in the dorsolateral prostate and downregulated in the ventral lobe (Figure 7). The reasons for lobe-specific effects observed in rodent models are poorly understood. For example, hyperprolactinemia induces hyperplasia in lateral prostate in rats (Van Coppenolle et al., 2000) while all lobes are affected in mice (Wennbo et al., 1997; Kindblom et al., 2002). Although the dorsolateral prostate is considered to be analogous to the peripheral zone of human prostate, this statement should be taken with caution as (i) human–rodent prostate analogies are primarily descriptive, and (ii) rodent do not spontaneously develop prostate tumors (Goffin et al., 2011). Based on the various readouts that we analyzed (weight, cell proliferation, gene expression profiling), the ventral lobe appeared to be the most sensitive to WS^®^ 1541. This is consistent with the findings of our former study related to lipidosterolic extract of *S. repens* (Bernichtein et al., 2015a). As shown in Table 1C (CD45+ cell clusters), the basal inflammatory status (vehicle group) of the Pb–PRL ventral prostate was two to threefold higher compared to the other lobes. In agreement, the absolute levels of expression of CD40lg and CTLA4 in the former (Ct ∼31) were much higher than in the latter (Ct ∼33–35). Therefore, it is likely that their downregulation in the ventral lobe correlates with the anti-inflammatory effect of WS^®^ 1541 in this lobe, while their upregulation in dorsal and ventral lobes may be a side effect independent of inflammation.

### Comparison With Other Drugs Used in the Treatment of BPH

Finasteride is a potent inhibitor of androgen signaling in the prostate, an effect that is mediated by inhibition of type II 5α-reductase which catalyzes the production of DHT, a high affinity androgen receptor (AR) ligand, from testosterone (Steers, 2001). In our study finasteride reduced the level of expression of the typical AR signaling target gene PSP94 (*p* = 0.027), indicating that the dose used was appropriate to produce pharmacological effects. Meanwhile, finasteride had a mild effect on prostate tissue growth (significant weight reduction of the ventral lobe only), and no significant reduction on cell proliferation whatever the readout. This result may indicate that androgens are not major drivers of prostate hypertrophy in this model. We nevertheless observed two adverse effects of finasteride: a marked increase of inflammatory cell infiltrates, and higher frequency of intraluminal mineralization.

Older studies involving rat models showed that complete abolition of androgen signaling by surgical castration led to rapid occurrence of prostatic inflammation, which was prevented by administration of exogenous androgens (Lundgren et al., 1984; Robinette, 1988). This indicates a strong opposing relationship between the level of androgen signaling and the regulation of non-bacterial inflammation. Accordingly, disruption of AR expression in luminal cells of the mouse prostate was shown to promote inflammation through the production of various cytokines including IL-1 and CCL2 (Zhang et al., 2016). Of interest, Vignozzi et al. (2013) observed that hypogonadism (i.e., low testosterone levels) was associated with a higher inflammatory score in human BPH tissue. In an animal model of prostatic inflammation resulting from high fat diet-induced hypogonadism, prostatic inflammation was completely reversed by testosterone supplementation (Vignozzi et al., 2012b). Furthermore, DHT pre-treatment of stromal cells derived from BPH patients abolished the secretion of several cytokines/chemokines induced by various pro-inflammatory stimuli (Vignozzi et al., 2012a). These observations indicate that androgen inhibition promotes prostatic inflammation, while androgenic stimulation has the opposite effect. Based on this evidence, it is not surprising that downregulation of androgenic signaling by finasteride increased prostatic inflammation in Pb–PRL mice. Among the pro-inflammatory genes screened in this study, finasteride increased expression of CCL4 (1.5-fold), CXCR4 (1.4-fold), and CD40lg (8-fold) in lateral prostate, and iNOS (twofold) and IL-2 (threefold) in ventral prostate (Figure 7). Other pro-inflammatory factors identified as AR-targets (e.g., IL-7, IL-8, IL-9, IL-12p75, bFGF, INF-γ, G-CSF, etc.) (Vignozzi et al., 2012a) may also contribute to this undesirable effect of finasteride. Indeed, a pro-inflammatory effect of finasteride has already previously been reported in men as the prevalence and extent of intraprostatic inflammation was higher in the finasteride than placebo arm of the Prostate Cancer Prevention Trial (Murtola et al., 2016).

*Corpora amylacea* are small round luminal hyaline masses that are believed to be related to epithelial cell desquamation and degeneration (Köseoglu, 2012). They are frequently seen in the benign acini of prostates of adult men and are supposed to be involved in the pathogenesis of BPH. The calcification of *C. amylacea* by progressive deposition of hydroxyapatite crystallites and mineralization with calcium forms prostate stones (or calculi). Obstruction of the acini by the latter may initiate a vicious circle favoring the increase in size of the stones resulting in occlusion of other acini by growing calculi. Such an occlusion mechanism might contribute to BPH (Köseoglu, 2012). Prostate stone incidence has been associated with inflammation, LUTS, chronic pelvic pain, and longer duration of symptoms (Shoskes et al., 2007; Hyun, 2018). Although the increased inflammation in the finasteride group may contribute to the prostate stone phenotypes, it remains unclear whether inflammation is a cause or the result of calculi formation (Köseoglu, 2012). Additional investigations are needed to elucidate this phenotype.

As mentioned, we recently evaluated in the Pb–PRL model the effects of another drug also used in BPH patients, namely Permixon^®^ (lipidosterolic extract of *S. repens*) (Bernichtein et al., 2015a). Although we used similar readouts as in this study, a direct efficacy comparison would have required both herbal drugs to be tested in the same study. The results are nevertheless consistent since the effect of WS^®^ 1541 on prostate weight, cell proliferation, and immune cell infiltrates nicely paralleled what was previously observed for Permixon^®^. However, with the exception of CCR7 and CXCL6, most of the cytokines/chemokines regulated by WS^®^ 1541 (see above) were not regulated by Permixon^®^, indicating drug-specific effects. Of interest, in both studies, the anti-inflammatory effect was accompanied by increased fibrosis as reflected by histological (picrosirius red) and molecular (COL1A1) readouts. Fibrosis can be regarded as a wound-healing process characterized by the activation and accumulation of myofibroblasts, which are transiently produced in many tissues as part of the normal wound response (Rodriguez-Nieves and Macoska, 2013). Although the etiology of fibrosis is uncertain, chronic inflammation has been raised as a probable candidate (Bushman and Jerde, 2016). Our data suggest that due to their anti-inflammatory effect, the wound-healing response has been initiated in mice treated with WS^®^ 1541 (or Permixon^®^) while in finasteride-treated animals the inflammatory process is still ongoing. If wound closure does not successfully occur, myofibroblasts do not receive mechanical signals to undergo apoptosis and continue to accumulate and deposit extracellular matrix, thereby replacing normal tissue with fibrotic tissue. Of note, it has been shown in mice that prostate fibrosis induced by inflammation of limited duration is partially reversible (Wong et al., 2014). Therefore, the phenotypes observed after 28 days of treatment with WS^®^ 1541 (or Permixon^®^) should not be regarded as a definitive. In BPH patients, fibrosis has been proposed to be associated with LUTS (Ma et al., 2012; Rodriguez-Nieves and Macoska, 2013; Bushman and Jerde, 2016) according to the fact that normal tissue is progressively replaced by collagenized tissue exhibiting higher stiffness and reduced elasticity (Rodriguez-Nieves and Macoska, 2013). Hence, antifibrotic therapeutic agents may be considered as a new approach for treating men with LUTS, especially in patients in whom other therapeutic regimens have not provided any improvement of symptoms.

## Conclusion

The concept that inflammation may promote BPH has been long suspected (Moore, 1937). However, it is only more recently that mechanistic studies have provided insights into the biochemical pathways underlying this association (see the section “Introduction”). We here report the potent efficacy of the herbal drug combination WS^®^ 1541 to reduce various histological hallmarks of BPH in a validated preclinical model, including tissue growth and stromal inflammation. The histological observations were supported by molecular profiling of prostates from mice treated with WS^®^ 1541 which exhibited reduced expression of several pro-inflammatory factors including cytokines/chemokines and their receptors, enzymes, and growth factors. These results support the beneficial role of WS^®^ 1541 in the management of BPH.

## Data Availability

All datasets generated for this study are included in the manuscript and/or the supplementary files.

## Author Contributions

NP, FB, EK, and VG conceived and designed the study. NP collected and assembled the data. NP, ER-G, EK, and VG analyzed and interpreted the data. NBD and SP provided intellectual input. NP, NBD, and VG wrote the manuscript. All authors approved the final version of the manuscript.

## Conflict of Interest Statement

This study was funded in part by Dr. Willmar Schwabe GmbH & Co. KG. EK is an employee of Dr. Willmar Schwabe GmbH & Co. KG. The funder was not involved in the study design, collection, analysis, and interpretation of data, the writing of this article or the decision to submit it for publication. The remaining authors declare that the research was conducted in the absence of any commercial or financial relationships that could be construed as a potential conflict of interest.

## Footnotes

1 http://www.tribvn.com/
